# Extramedullary Myeloid Tumor Involving the Pancreas: A Case Report and Review of the Literature

**DOI:** 10.4274/tjh.2012.0166

**Published:** 2014-09-05

**Authors:** Semra Paydaş, Hakan Özdoğu, Meral Günaldı, Veysel Haksöyler, Arbil Açıkalın, Melek Ergin

**Affiliations:** 1 Çukurova University Faculty of Medicine, Department of Medical Oncology, Adana, Turkey; 2 Çukurova University Faculty of Medicine, Department of Medical Hematology, Adana, Turkey; 3 Çukurova University Faculty of Medicine, Department of Internal Medicine, Adana, Turkey; 4 Çukurova University Faculty of Medicine, Department of Medical Pathology, Adana, Turkey

**Keywords:** Extramedullary myeloid tumor, Pancreas, allogeneic stem cell transplantation

## Abstract

Extramedullary myeloid tumors (EMMTs) are the tumors of myeloid cells. These tumors may occur in all of the organs of the body, but some localizations are rare. Pancreatic involvement of EMMTs is a rare entity. Here we report a case of EMMT of the pancreas 4 years after allogeneic stem cell transplantation and we review the existing data about EMMTs involving the pancreas.

## OZET

Extramedüller miyeloid tümörler (EMMT) miyeloid hücrelerin neoplazileridir. Bu tümörler bütün organlarda oluşabilir fakat bazı lokalizasyonlarda EMMT saptanması nadirdir ve pankreasın EMMT ile tutulumu da nadirdir. Burada allogeneik kök hücre naklinden 4 yıl sonra gelişen pankreas EMMT’ü sunulmuş ve mevcut bilgi gözden geçirilmiştir.

## INTRODUCTION

Granulocytic sarcoma is the tumor of immature myeloid cells. It was first defined by Burns about 100 years ago [1]. There are many synonyms for this entity but the most recent is “extramedullary myeloid tumor” (EMMT) [2]. It may be isolated or may accompany myeloid neoplasias, including acute myeloblastic leukemia (AML), chronic myeloid leukemia (CML), and myeloproliferative/myelodysplastic neoplasias [3]. An important and interesting point for EMMT has been its development after allogeneic stem cell transplantation [4]. The incidence of EMMT in such cases is highly variable, but an unchanged reality is the poor prognosis of malignancies accompanying EMMTs. The management is systemic chemotherapy according to the underlying malignancy in addition to local treatments including surgery and/or radiation therapy. Nearly all tissues and organs may be involved in an EMMT. The most commonly involved organs are the skin, lymph nodes, soft tissues, bone, and periosteum; less frequently, other tissues or organs have also been reported to be involved in EMMT. However, some organ involvements are rare. Pancreatic EMMT is rare; so far, 11 cases, 2 after allogeneic stem cell transplantation, have been reported. Here we report a case of EMMT of the pancreas presenting as jaundice and mimicking pancreatic cancer, and the literature is reviewed.

## CASE PRESENTATION

A 37-year-old man was admitted to the hospital at December 2010 with fatigue, abdominal pain, and a 10-kg weight loss over 6 months. The pain was localized to the epigastrium and increased following food intake. Upper endoscopic examination 4 months prior to admission showed no abnormality. One month later he noted dark urine and jaundice. Abdominal ultrasonography showed a hilar mass of the pancreas. Additionally, there was intra/extrahepatic bile duct dilatation, hydropic gallbladder, and splenomegaly. Upper abdominal MRI and MR angiography showed a mass lesion localized at the head of pancreas, invading the superior mesenteric vein and bilateral renal veins with intra/extrahepatic bile duct dilatation. In October, a cholecystectomy was performed. Frozen section biopsy taken from the pancreas revealed chronic pancreatitis. After surgery, he was referred to our department and was hospitalized.

He had a past medical history of intermediate-risk AML 5 years prior to admission. He had received 1 course of induction treatment (cytosine arabinoside, 7 days, plus idarubicin, 3 days) and 3 courses of postinduction treatment (high-dose cytosine arabinoside), and then an allogeneic stem cell transplant from his sister 4 years prior to admission. The conditioning regimen was Bu 6.4/Flu 120/ATG 20. Cyclosporine had been given for 11 months after transplantation with no subsequent immunosuppressive treatment. His family and social histories were unremarkable. Informed consent was obtained.

Vital signs were within normal limits upon physical examination. There was jaundice, ascites, and abdominal scars associated with surgery. The liver and spleen were palpable 2 and 3 cm below the right and left costal margins, respectively, and there was (+++) pretibial edema.

Laboratory findings were Hb of 11.1 g/dL, WBC 4x109/L, platelet count 194x109/L, blood urea nitrogen/creatinine 3.9/0.4 mg/dL, total/direct bilirubin 6.4/1.9 mg/dL, lactate dehydrogenase 546 IU, aspartate aminotransferase/alanine aminotransferase 48/87 IU, alkaline phosphatase 2348 IU, total protein/albumin 5.4/1.9 mg/dL, ferritin 1397 ng/mL, PT-INR 1.3, and thyroid function tests within normal limits. Hepatitis antibodies for HBV and HCV were found to be negative. Ascites cytology was negative for tumor cells. Other tumor markers were CEA, 1.1; CA19-9, 9.26; AFP, 10; and CA-125, 352 IU.

**Figure 1:**
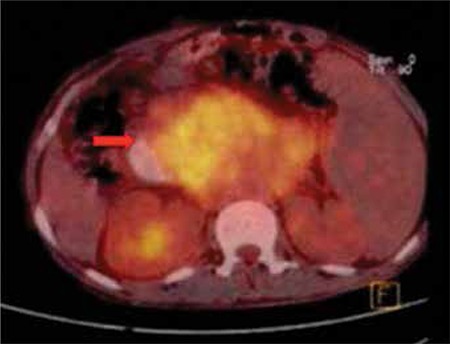
PET/CT showing pancreas and surrounding tissue invasion by extramedullary myeloid tumor.

PET/CT showed a huge mass involving the pancreas and invading surrounding structures (Figure 1).

**Figure 2:**
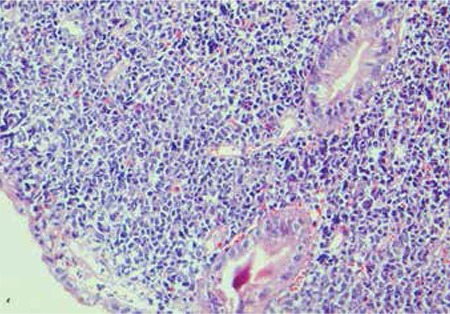
Biopsy taken from duodenal mass showing extramedullary myeloid tumor.

**Figure 3:**
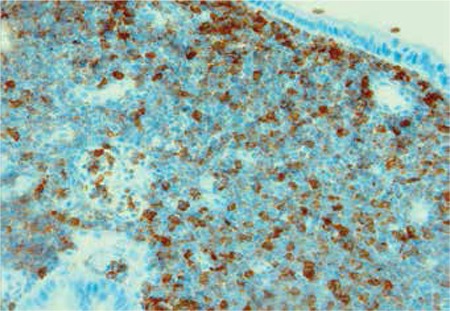
Strong myeloperoxidase staining in tumor.

Upon follow-up, the most important problem of the patient was dyspeptic complaints. Upper gastrointestinal endoscopic examination was repeated and enterogastric reflux, F1 varices, portal hypertensive gastropathy, and a duodenal mass obstructing the lumen and diverticula were detected. Histopathological exam of the biopsy taken from the duodenal mass revealed myeloid sarcoma (Figure 2) with strongly positive myeloperoxidase (Figure 3). Molecular study of the origin of this tumor showed that 98% was patient-origin and 2% was donor-origin. There was no evidence of leukemic infiltration in the bone marrow and biopsy was normocellular. Pancreatic resection and radiation therapy were deemed impossible due to the very large tumor invading vital structures. Salvage FLAG-IDA (fludarabine–cytosine arabinoside–idarubicin–G-CSF) chemotherapy was given, but the patient died of neutropenic sepsis 8 months after his first gastrointestinal symptoms. Clinical presentations, bone marrow status at the beginning, treatment approaches and responses, and the last status are presented in Table 1.

## DISCUSSION AND REVIEW OF THE LITERATURE

Nearly all of the organs have been reported to be involved with EMMTs. The most commonly involved organs are the skin, lymph nodes, soft tissues, bone, and periosteum. The testes, kidneys, orbit, ovaries, uterus, bladder, gingiva, and stomach are the other sites involved in EMMTs [3]. Pancreatic involvement has been reported, but it is a relatively uncommon localization for EMMTs. Eleven cases (2 after transplant) of pancreatic EMMT were reported between 1987 and 2011 [5,6,7,8,9,10,11,12,13]. In nontransplant patients, the age was between 31 and 75 years, and 7 of them were female. The most common presenting symptoms of these patients were abdominal/epigastric pain and jaundice. Weight loss, fever, and acute abdomen are less likely reported symptoms. Four of these 9 cases had no known history of AML or other myeloid malignant disorder and these cases were clinically mimicking pancreatic cancer. At the beginning, fine needle aspiration or open biopsies reported chronic inflammation or high-grade lymphoma. Myeloid origins of these tumors were detected after systemic evaluation or careful histopathological examination by myeloperoxidase and chloroacetate esterase. Four patients had newly diagnosed AML or bone marrow biopsy was done due to peripheral cytopenias, and blastic infiltration was detected at rates between 6% and 78%. In 1 case, there was AML history 8 months prior, but there was no evidence of bone marrow infiltration when the patient presented with jaundice. Among these 9 cases, 7 were treated by AML-type chemotherapy (regimens involving anthracycline plus Ara-C), while 2 did not receive chemotherapy in spite of recommendations. Eight of these 9 patients achieved complete response after chemotherapy and/or surgical approach. Survival times were variable; 1 patient died 45 days after diagnosis, while 1 was living 49 months after allotransplantation.

EMMTs are not rare after allogeneic transplantation and their most common localizations are the breast, gastrointestinal tract, skin, spine, central nervous system, and testes [4]. Looking at the literature, pancreatic involvement after transplantation is very rare. We found only 2 cases of EMMT with pancreatic involvement after allogeneic transplantation. One of these 2 patients had AML, and relapse in the pancreas developed 2 years after allogeneic transplantation. Only chemotherapy was given and the patient was reported to be in remission 89 months after EMMT development [4]. The second case involved CML and a pancreatic mass developed 15 months after transplantation while the bone marrow was in remission. Pancreatic resection was done and immunosuppressive drugs were stopped, but bone marrow relapse developed and the patient died 4 months later [14]. In general, the biology of pancreatic EMMT is not different from other sites of EMMTs, but delayed diagnosis may be the cause of the bad outcome of this entity. In principle, treatment includes local treatment as well as systemic antineoplastic AML-type treatment. 

Pancreatic involvement of EMMT developed in our case after allogeneic transplantation. At the beginning, pancreatic cancer had been suspected in the department of surgery. However, the history of AML and allotransplantation suggested EMMT. Histologic confirmation of this entity was made by duodenoscopic mass biopsy. Although the first endoscopic exam was negative when the patient was admitted for pancreatic mass, the endoscopic exam was repeated and biopsy of the then-detected mass showed myeloid sarcoma; this was confirmed by myeloperoxidase stain. There was no evidence of bone marrow relapse in our case. 

In conclusion, although rare, EMMT may be seen in the pancreas and may mimic pancreatic cancer. Pancreatic EMMT may be seen either in isolation or accompanying myeloid malignancies, and also following allogeneic stem cell transplantation.

**Conflict of Interest Statement**

The authors of this paper have no conflicts of interest, including specific financial interests, relationships, and/ or affiliations relevant to the subject matter or materials included.

## Figures and Tables

**Table 1:**
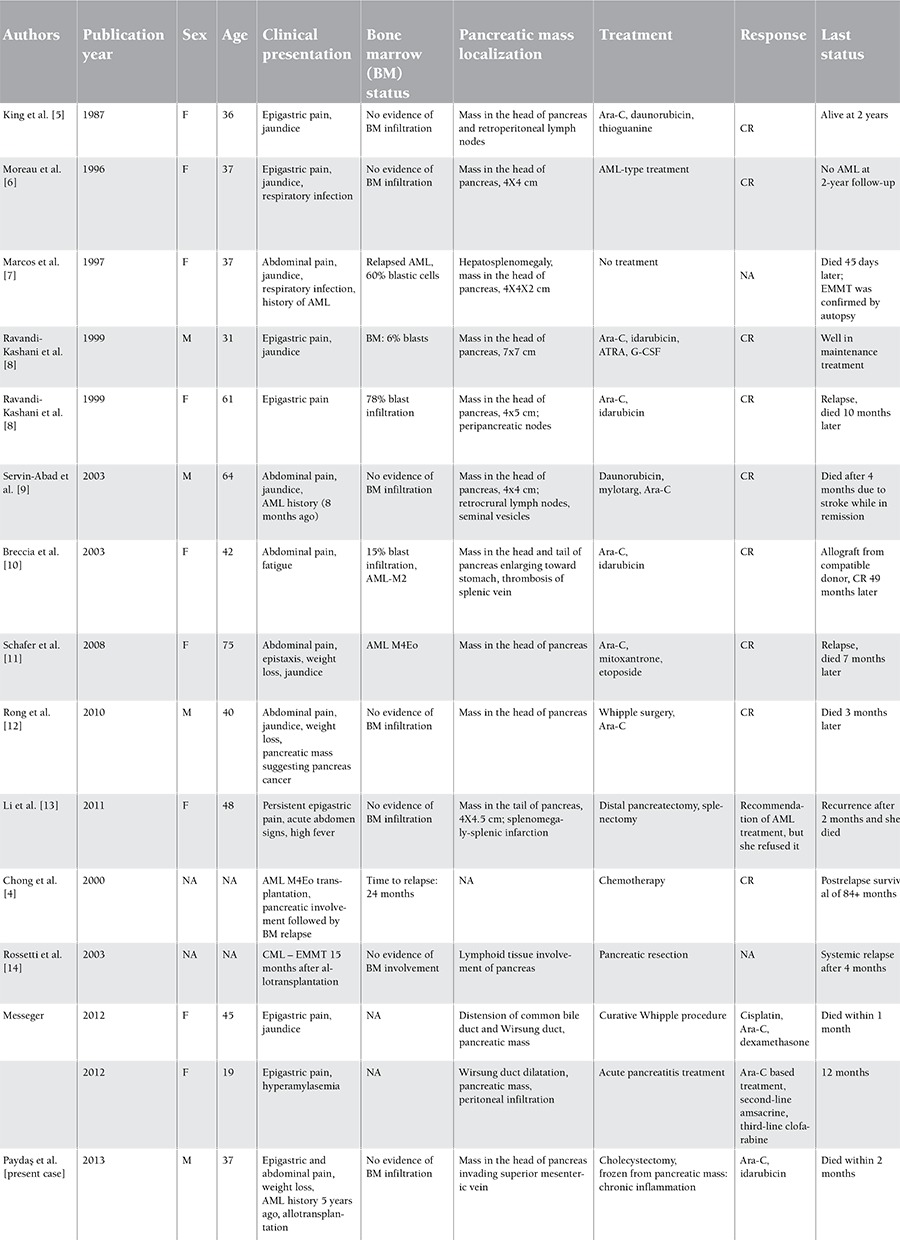
Clinical presentation, treatment approaches, and outcome in cases of pancreatic EMMT (CR: complete response, NA: not assessed).
